# Multi-Omics Insights into Microbial Interactions and Fermented Food Quality

**DOI:** 10.3390/microorganisms13122679

**Published:** 2025-11-25

**Authors:** Jiayi Ji, Xinyue Jiang, Panpan Song, Qi Yang, Mengying Sun, Zhihui Dong, Yi Lu, Shaohua Dou, Liang Dong

**Affiliations:** 1 College of Life and Health, Dalian University, Dalian 116622, China; 2 Liaoning Provincial Engineering Research Center for Marine Microbiology, Dalian 116622, China; 3 Dalian Key Laboratory of Animal Immunology, Dalian 116622, China

**Keywords:** microbial communities, fermented foods, multi-omics technologies, flavor compounds

## Abstract

The quality, flavor, and functional attributes of fermented foods are intrinsically shaped by the composition and metabolic dynamics of their microbial communities. This systematic review explores the structural organization, successional patterns, and mechanistic roles of these communities in influencing food quality, with a specific focus on core functional groups—including lactic acid bacteria (LAB), yeasts, and molds—and their interplay through key metabolic pathways. By integrating multi-omics approaches, such as metagenomics and metabolomics, we elucidate the underlying relationships between microbial activity and the formation of volatile flavor compounds, nutritional metabolites, and bioactive substances. These insights offer a scientific basis for the targeted regulation and functional enhancement of fermented food products.

## 1. Introduction

Fermentation is a fundamental biological process in which microorganisms enzymatically decompose complex organic macromolecules into simpler metabolites. As an integral component of the global carbon cycle, fermentation also represents one of humanity’s oldest and most vital biotechnologies for producing and preserving foods with improved nutritional and sensory properties [1,2]. Microbial communities involved in fermentation are inherently dynamic; however, they frequently exhibit progressive stabilization over time, a phenomenon largely governed by interspecies interactions [3,4,5]. Key ecological processes—including community succession, structural stability, and functional evolution—are strongly influenced by mechanisms such as competition, predation, physiological adaptation, and gene transfer [5,6]. A representative example comes from low-temperature soy sauce mash fermentation, where sequential inoculation of the halophilic bacterium *Tetracoccus* and the yeast *Variabilis* enhanced the production of key flavor compounds through microbial synergy, illustrating how interaction-guided strategies can improve product quality [7]. Similarly, solid-state co-fermentation of moringa seeds using *Aspergillus oryzae* and *Aspergillus niger* significantly increased the concentration of bioactive components. This cooperative process improved the utilization of total phenolics by nearly 50% compared to monoculture fermentation, substantially strengthening the antioxidant activity of the final product [8]. Therefore, elucidating the mechanisms underlying microbial interactions has become a central research priority, with important implications for the rational design of microbial consortia and precision control of fermentation systems.

**Figure 1:**
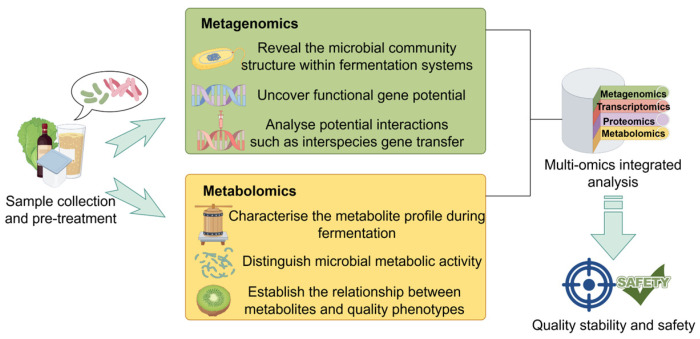
Multi-omics research strategy for analyzing microbial interactions in fermented foods.

In naturally fermented foods, quality attributes are largely governed by functional interactions within core microbial communities [9,10,11]. However, traditional fermentation processes often rely on spontaneous microbial succession in open environments, where community stability is highly susceptible to variations in raw material composition, environmental conditions, and operational practices. This inherent variability poses significant challenges to achieving consistent product quality and standardization [12,13,14,15]. Moreover, conventional analytical approaches based on single-omics technologies—such as metagenomics—have notable limitations: they often fail to detect rare functional taxa, cannot resolve metabolic activity at the species level, and provide limited insight into the molecular basis of microbial interactions. As a result, correlating community structure with functional outputs remains a major challenge [16,17]. As illustrated in Figure 1, To systematically decipher microbial interaction networks and enable targeted regulation, integrated multi-omics approaches are essential. When combined with emerging tools such as synthetic microbial community engineering, these strategies offer a promising pathway to advance fermentation from an experience-based practice toward a mechanism-driven and predictable process [18,19].

Although microbial interactions are widely recognized as critical determinants of fermented food quality, comprehensive reviews that systematically summarize their underlying mechanisms and corresponding regulation strategies remain scarce. This review begins by synthesizing current knowledge on the composition, dynamics, and interaction patterns of microbial communities in fermented foods. Special emphasis is placed on how microorganisms collectively influence the formation of flavor, texture, and safety attributes through metabolic cooperation and competition. In contrast to earlier reviews, this work highlights the application of emerging methodologies—such as multi-omics integration, synthetic microbiome engineering, and genome-scale metabolic modeling—and assesses their potential in clarifying interaction mechanisms and optimizing product quality. Furthermore, we discuss how interdisciplinary collaboration can facilitate the transition of fermentation processes from empirical tradition to precision biotechnology. Ultimately, this analysis aims to provide a theoretical foundation for driving the fermentation industry toward standardization, functional enhancement, and sustainable development.

## 2. Microbial Community Structural Characteristics in Fermented Foods

### 2.1. Major Microbial Groups and Their Functions

The microorganisms involved in food fermentation processes are primarily derived from two sources: firstly, through the intentional addition of fermentation starters (such as *koji* mold or defined starter cultures); and secondly, from the indigenous microbiota naturally associated with raw materials or present in the production environment [20,21,22]. Although traditional fermentation systems typically harbor complex and diverse microbial communities—including yeasts, filamentous fungi, lactic acid bacteria, and acetic acid bacteria—only a limited number of key taxa tend to dominate at different stages of fermentation [23,24,25]. These core microbial groups drive the fermentation process, regulating the synthesis and accumulation of flavor compounds and functional constituents through their metabolic activities, thereby directly shaping the final quality and distinctive attributes of fermented foods.

The composition of core microorganisms varies considerably across different types of fermented products. In fermented vegetables, dominant taxa include LAB, *Leuconostoc*, *Lactococcus*, *Weizmannia*, and *Lactobacillus* [26]. These microorganisms significantly reduce product pH, inhibit spoilage bacteria growth, and create a distinctive tart flavor by producing organic acids such as lactic acid and acetic acid, alongside bacteriocins. Cereal-based fermentations commonly involve species such as *Lactobacillus plantarum*, *Lactobacillus mesenteroides*, *Penicillium* spp., *Saccharomyces cerevisiae*, *Enterobacter faecalis*, *Bacillus amyloliquefaciens*, *Lactococcus lactis*, *Hansenula moniliformis*, and *Bacillus salinus* [27]. These microorganisms synthesize organic acids, ethanol, esters, and polysaccharides, collectively determining the product’s acidity, alcoholic aroma, flavor complexity, and viscous texture. Beverage fermentations are frequently driven by *Aspergillus oryzae*, *Saccharomyces cerevisiae*, *Bacillus pasteurii*, *Acetobacter xylinum*, *Acetobacter glucosum*, and *Bacillus subtilis* [27]. Their metabolic activities produce alcohol, acetic acid, amino acids, and aromatic esters, directly influencing the beverage’s alcohol content, acidity, umami, and fruity undertones. In fermented meat products, common microorganisms include *Lactobacillus sakei*, *Lactococcus lactis* subsp. *lactis*, *Lactobacillus plantarum*, *Carnobacterium carnous*, *Leuconostoc gelidum*, *Bifidobacterium bifidum*, *Enterococcus durans*, *Enterococcus faecalis*, *Enterococcus hirae*, *Bacillus subtilis*, *Lactobacillus divergens*, *Lactobacillus carnis*, *Enterococcus cecorum*, and *Bacillus lentus* [27,28]. These microorganisms lower pH by producing lactic acid, hydrogen peroxide, and antimicrobial peptides, while simultaneously forming volatile flavor compounds such as ketones and aldehydes. This endows the meat products with a stable shelf life and distinctive fermentative aromas. Dairy fermentations typically rely on a core microbiota comprising *Lactobacillus delbrueckii* subsp. *bulgaricus*, *Lactococcus lactis*, *Lactobacillus acidophilus*, *Lactobacillus cremoris*, *Streptococcus thermophilus*, *Lactobacillus casei*, *Lactobacillus paracasei*, *Lactobacillus kefiri*, *Lactobacillus caucasicus*, *Penicillium camemberti*, *Acetobacter loevanii*, *Penicillium roqueforti*, *Saccharomyces cerevisiae*, and *Saccharomyces boulardii* [27]. These collaborate to produce lactic acid, ketones, acetaldehyde, and extracellular polysaccharides, critically influencing curd texture, acid-sweet balance, buttery aroma, and velvety mouthfeel.

Given the critical influence of microbial composition on the quality, safety, and shelf life of fermented foods, in-depth analysis of the physiological, metabolic, and genetic features of these core microorganisms has become essential for achieving standardized and precision-controlled industrial production.

### 2.2. Community Dynamics

Fermented foods represent an ideal model for investigating the dynamic succession of microbial communities. Facilitated by open production conditions and nutrient-rich matrices, microorganisms undergo continuous growth, competition, and orderly progression throughout the fermentation process [29]. This succession is governed by three primary factors: the initial microbial inoculum, environmental conditions (e.g., temperature, pH, water activity), and specific fermentation parameters. Together, these factors drive a functionally phased succession of microorganisms that ultimately shapes the flavor, texture, and safety of the final product.

Comparative analysis of various fermentation systems reveals that microbial succession follows shared ecological principles while displaying pathway diversity depending on raw materials and process design. In the solid-state, multi-stage fermentation system of *baijiu*, microbial succession exhibits a clear sequential pattern. The initial phase is driven by a pioneer community including fungi such as *Saccharomyces*, *Aspergillus*, and *Mucor*, along with bacteria like *Acetobacter* and *Lactobacillus*, which hydrolyze starch and proteins to generate substrates for subsequent metabolic steps [30]. As fermentation progresses to mid- and late stages, the fungal community shifts with increased abundance of *Fusarium* species, while the bacterial niche becomes dominated by *Lactobacillus*. Their metabolic outputs—such as ethanol and higher alcohols from yeasts, and organic acids from lactic acid bacteria—act synergistically to develop flavor complexity [31]. By the final stage, lactic acid bacteria emerge as the dominant group (reaching up to 96.28% relative abundance), with their proliferation strongly correlated with the accumulation of key flavor compounds such as esters and n-propanol.

In contrast, kombucha fermentation demonstrates a linear succession driven by metabolite transfer in a liquid system. During the initial phase, yeasts convert sucrose to ethanol; in the mid-phase, acetic acid bacteria oxidize ethanol to acetic acid and form a symbiotic cellulose biofilm (SCOBY) that modulates the microenvironment; by the late stage, lactic acid bacteria become increasingly active, further accumulating organic acids and flavor metabolites, thereby establishing a sequential “sugar → alcohol → acid” metabolic cascade [32].

The natural fermentation of kefir exhibits clear temporal dynamics in its microbial succession. During the initial stage, the bacterial community is dominated by *Lactococcus* (approximately 62%) and *Bacteroides*, while the fungal community is primarily composed of *Aspergillus* and *Cordyceps*. As fermentation progresses to 24 h, the dominance of *Lactococcus* further increases (reaching about 72.6%), accompanied by a notable rise in *Pseudomonas*. Within the fungal community, the relative abundance of *Aspergillus* declines, while that of *Cordyceps* increases to 24%. As the core functional group, *Lactococcus* drives lactose fermentation and acid production. Its sustained enrichment, along with complementary shifts in the fungal community, collectively shapes the distinctive flavor and functional properties of kefir [33].

Similarly, the maturation of cheese involves distinct phases of microbial succession. The initial stage is dominated by lactic acid bacteria, which acidify the medium and coagulate the milk. In later stages, surface yeasts, molds such as *Penicillium*, and bacteria such as *Bacillus licheniformis* contribute to the degradation of proteins and lipids, collectively forming the characteristic flavor profile of the cheese [34]. In contrast, Eastern fermented vegetables such as Korean kimchi undergo rapid acid and gas production during the initial phase, driven mainly by heterofermentative lactic acid bacteria including *Leuconostoc* species. As fermentation proceeds, homofermentative lactic acid bacteria—primarily *Lactobacillus* species—become dominant, facilitating acid accumulation and achieving a harmonized flavor balance [35].

Collectively, these examples illustrate that successful fermentation relies fundamentally on the coordinated action of key functional microorganisms within appropriate spatial and temporal contexts. Succession is driven by a combination of interspecies interactions—such as nutrient competition, metabolite-mediated inhibition, and cross-feeding—coupled with gradients in environmental conditions. A deeper understanding of microbial succession patterns not only helps elucidate the quality formation mechanisms in traditional fermented foods, but also provides a theoretical basis for improving product consistency through optimized inoculation strategies and environmental control. Viewing fermented foods as dynamic microbial ecosystems represents a crucial scientific approach for advancing traditional fermentation from empirical practice toward controlled, standardized production.

## 3. Metabolite Synthesis Mediated by Microbial Interactions

Within the complex ecosystem of fermented foods, diverse microbial populations collectively shape the metabolic profile of the final product through various forms of interactions. As summarized in Figure 1, these interactions include mutualism, competition, amensalism, and commensalism. They influence microbial community structure through direct mechanisms (e.g., physical contact, signal transduction) or indirect means (e.g., modification of the microenvironment), thereby activating diverse biosynthetic pathways related to vitamin synthesis, peptide conversion, and accumulation of bioactive compounds. These processes collectively enhance the nutritional and flavor attributes of fermented foods [36].

### 3.1. Mutualism

**Figure 2:**
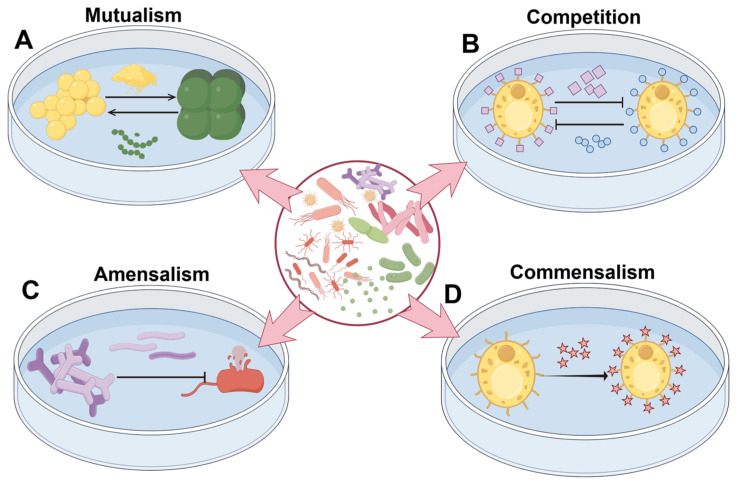
Forms of microbial interactions. (**A**) Mutualism: Two microorganisms mutually provide substances essential for survival (such as metabolites and enzymes), thereby promoting each other’s growth and forming a stable symbiotic relationship; (**B**) Competition: Two microorganisms compete for limited resources (such as nutrients or space), with one gaining the upper hand by secreting inhibitory substances or proliferating rapidly, thereby suppressing the growth of the weaker counterpart; (**C**) Amensalism: One microorganism releases harmful substances (such as toxins or enzymes) to inhibit the growth of another microorganism, while remaining unaffected itself; (**D**) Commensalism: One microorganism benefits (e.g., by gaining shelter or nutrients), while the other remains unaffected.

Mutualism represents a synergistic form of interdependence among microorganisms in fermentation systems (Figure 2A). Its core mechanism lies in metabolic cross-feeding, whereby intermediates or signaling molecules produced by one microorganism are utilized by another, activating specific metabolic pathways and improving the synthesis efficiency of target compounds [37]. The exchanged substances range from basic metabolites such as sugars, organic acids, amino acids, and vitamins, to functional molecules like quorum-sensing signals and siderophores.

A notable example comes from grape juice fermentation studies: using synthetic ecology approaches, researchers successfully established an engineered mutualism between *Saccharomyces cerevisiae* (BY4742Δthi4) and *Lactobacillus plantarum* (IWBT B038) [38]. In the presence of carbon sources such as glucose and fructose, these two organisms exhibit mutually enhanced growth. Lactic acid metabolized by *L. plantarum* serves as an auxiliary carbon source for *S. cerevisiae*, while the yeast may in turn support the growth of lactic acid bacteria by supplying B vitamins. Importantly, even low concentrations of lactic acid produced by *L. plantarum* enhance the synthesis of ethyl acetate—a contributor to fruity aroma in wine—by *S. cerevisiae,* while significantly suppressing the formation of undesirable higher alcohols, thereby improving the overall wine quality [39].

This example illustrates that understanding and leveraging natural mutualistic relationships can not only stabilize fermentation processes but also enable targeted modulation of sensory properties, offering both theoretical and practical pathways for the precision design of fermented foods.

### 3.2. Competition

Competition in fermentation systems mainly involves contestation over nutrients and ecological niches (Figure 2B) [40]. Although some microorganisms can transiently inhibit others by secreting antimicrobials such as organic acids or bacteriocins, this suppression is often temporary. Within inhibited populations, tolerant strains frequently emerge, eventually outcompeting previously dominant groups through more efficient resource utilization.

In yogurt fermentation, for instance, *Streptococcus thermophilus* and *Lactobacillus delbrueckii* subsp. *bulgaricus* exhibit mutualistic symbiosis, yet under certain conditions they also compete for carbon or nitrogen sources. In Cheddar cheese, *S. thermophilus* mitigates nitrogen limitation in *Streptococcus lactis* by releasing peptides and amino acids via proteolytic activity, highlighting nitrogen as a key limiting resource [41]. Further studies reveal that during the late maturation phase of cheese, inoculated *Lactococcus lactis* starter cultures can dominate, accounting for 70–87% of the microbial population, demonstrating their superior competitiveness and adaptability, which inevitably reshapes nitrogen resource distribution across the system [42].

Overall, microbial competition acts as a dynamic ecological driver that not only directs community succession but also critically influences final product quality. However, most current studies remain descriptive; future work should integrate real-time monitoring and multi-omics technologies to dissect the molecular mechanisms underlying competitive interactions, such as signaling and environmental adaptation. Regulating competition intensity to steer community succession may offer novel strategies for flavor customization and process standardization.

### 3.3. Amensalism

Amensalism occurs when one microorganism adversely affects another by releasing inhibitory metabolites (e.g., bacteriocins, organic acids), without being affected itself (Figure 2C). This interaction helps regulate metabolic networks and reduces the accumulation of harmful metabolites such as biogenic amines and mycotoxins, thereby ensuring product safety and quality.

In traditional solid-state fermentation of *baijiu*, bacilli isolated from *Daqu* inhibit *Streptomyces sampsonii*—the main producer of geosmin—effectively eliminating earthy off-odors through bioantagonism [43]. In meat products, LAB such as *Lactobacillus sakei* CTC494 rapidly dominate and produce sakacin K, a bacteriocin that significantly suppresses *Listeria* monocytogenes. Studies show that when LAB reach high cell densities, they also effectively inhibit *Listeria*, especially under low-temperature conditions [44]. Similarly, during kimchi fermentation, LAB produce lactic acid that acidifies the environment, effectively restraining the growth of contaminating *E. coli* and ensuring normal fermentation progression [45,46,47].

These cases illustrate that successful microbial inhibition in food systems depends on the interplay of strain fitness, environmental conditions, and metabolic activity.

### 3.4. Commensalism

Commensalism is a classic microbial interaction in which one organism benefits while the other remains unaffected (Figure 2D). This unidirectional benefit makes it a suitable strategy for precision modulation: the introduction of specific functional microbes can enhance product quality without disrupting the native fermentative community.

In wine production, for example, the interaction between *Saccharomyces cerevisiae* and *Staphylococcus vitulinus* during malolactic fermentation exemplifies commensalism. *S. vitulinus* enhances the growth of *S. cerevisiae* without inhibiting lactic acid bacteria, leading to shorter fermentation cycles and improved process efficiency [48].

Soy sauce fermentation further demonstrates the role of commensalism in microbial succession and flavor development. In the initial phase, halophilic tetracocci rapidly metabolize sugars to produce lactic acid, lowering the system pH and creating a favorable environment for acid-tolerant yeasts such as *Saccharomyces rouxii* [49]. Simultaneously, the proteolytic activity of halophilic bacteria releases free amino acids, which serve as a nitrogen source for *Saccharomyces* spp., including *S. rouxi*, supporting the synthesis of characteristic aroma compounds such as 4-ethylguaiacol [50]. This case confirms that commensalism not only drives microbial succession but also serves as an effective ecological strategy for targeted flavor enhancement.

## 4. Quality Regulation in Fermented Foods Driven by Microbial Interactions

**Figure 3:**
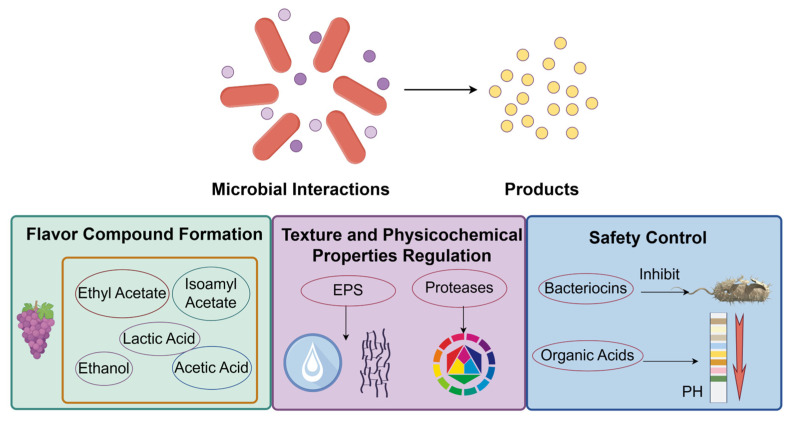
Microbial Interactions and Their Functional Metabolites in Fermented Foods.

Based on a comprehensive understanding of the relationships between microbial interactions and fermented food quality, this section examines how such interactions regulate three fundamental quality attributes: flavor, texture, and safety. As depicted in Figure 3, microbial communities collectively drive the synthesis and transformation of key metabolites through interconnected metabolic networks, thereby directly shaping the final quality profile of fermented products. The following discussion systematically addresses the formation of flavor compounds, the modulation of texture and physicochemical properties, and strategies for ensuring microbial safety—highlighting the essential role of microbial interactions in defining the overall quality of fermented foods.

### 4.1. Formation of Flavor Compounds

#### 4.1.1. Typical Flavor Compounds

Esters represent a pivotal class of flavor compounds in fermented foods, widely distributed across products such as fermented fish, beer, dairy items, and wine [51,52,53,54]. Beyond imparting desirable floral and fruity notes, they effectively mask undesirable odors derived from fatty acids and amines [55]. Ethyl esters containing medium- and short-chain fatty acids are particularly valued for their pleasant aroma profiles. Studies indicate that microbial esterases play a central role in ester biosynthesis [56,57,58]. In recent years, LAB, recognized for their GRAS (Generally Recognized as Safe) status, have attracted significant research attention. Their esterases, when employed in fermentation, can accelerate process kinetics, enhance ester yields, and refine product flavor. Paiva et al. reported that ethyl esters can be synthesized via pathways such as acidolysis and transesterification, mediated by lactic acid bacteria, yeasts, or molds [59]. For instance, during alcoholic fermentation, the esterase activity of *Lactobacillus helveticus* Lac34 reduces the formation of short-chain ethyl esters like ethyl acetate [60], whereas supplementation with *Lactobacillus plantarum* promotes the accumulation of esters such as ethyl lactate, ethyl acetate, and amyl acetate [61].

Ethanol, another key flavor compound, is mainly generated through heterofermentative pathways in lactic acid bacteria, where glucose is metabolized into lactic acid, ethanol, and carbon dioxide. With its distinct olfactory and trigeminal effects, ethanol enhances flavor perception at appropriate concentrations. In fermented dairy products such as yogurt, ethanol levels typically range between 0.2 and 9.9 mg/kg [62]. Research has shown that fermentation by *Streptococcus thermophilus* IMAU80842 or *Lactobacillus bulgaricus* IMAU20401 individually, or in co-culture, yields not only ethanol but also other detectable alcohols including 1-hexanol and 1-heptanol. Notably, ethanol concentration continues to increase during storage, peaking at 8.13–10.99%, while acetaldehyde levels concurrently decline [63,64].

The sour taste in fermented foods is largely shaped by acetic acid, glucuronic acid, and related derivatives. Acetic acid contributes a sharp and pungent acidity, whereas glucuronic acid imparts a milder, more refreshing sourness [65]. In kombucha, for example, yeasts preferentially metabolize fructose to produce ethanol via glycolysis, while acetic acid bacteria oxidize glucose at the C-6 position to yield glucuronic acid and further transform ethanol into acetic acid [66]. These organic acids not only define the product’s sour character but also underpin its preservative properties [67].

Kefir illustrates how multiple flavor compounds arise from microbial synergy. This fermented milk beverage, produced through symbiotic culture of lactic acid bacteria and yeasts, develops its distinctive flavor from a complex metabolic network: lactic and acetic acids provide refreshing sourness, while ethanol (typically 0.08–2.0% *v*/*v*) and carbon dioxide contribute subtle wine-like notes and effervescence. Volatile compounds such as acetaldehyde collectively shape a flavor profile characterized by a recognizable “yeasty” aroma [68,69,70,71]. Microbial interactions not only directly synthesize flavor compounds but also modulate precursor turnover, thereby shaping the overall sensory outcome.

In summary, volatile compounds—including esters, alcohols, and organic acids—play a decisive role in defining the flavor of fermented foods. Esters enhance sensory appeal through fruity and floral notes while mitigating off-flavors; ethanol and acetaldehyde, among others, determine flavor intensity and consumer acceptance when present in balanced concentrations. Current evidence confirms that modulating microbial community structure and enzymatic activities can redirect metabolic flux and influence the accumulation of these flavor compounds. This understanding offers a solid foundation for optimizing traditional fermentation processes and developing novel fermented products with tailored flavor profiles.

#### 4.1.2. Multi-Pathway Collaborative Flavor Orientation Regulation Strategy

During fermentation, bacteria and fungi metabolize carbohydrates, proteins, and lipids. This process not only supplies energy for microbial growth but also yields a wide array of volatile compounds that confer distinctive aromas and flavors to fermented foods. These volatiles play a fundamental role in defining the sensory properties and overall acceptability of the final product.

The flavor-forming potential of a fermentation system is governed by the interplay between microbial composition, process parameters—such as temperature, pH, and duration—and substrate characteristics. In the initial phase, microorganisms rapidly colonize the substrate surface, producing primary metabolites including amino acids and vitamins while obtaining energy for proliferation [72]. As fermentation progresses into the mid-to-late stages, microorganisms enter exponential and stationary growth phases, during which they synthesize substantial quantities of secondary metabolites. These compounds are closely associated with the functional and sensory attributes of the food. Among them, volatile metabolites—organic compounds with molecular weights typically between 50 and 200 Da—constitute only a minor proportion of total microbial metabolites, yet they exert a decisive influence on the final quality of fermented products [73]. As summarized in Table 1, the principal metabolic pathways contributing to volatile formation include carbohydrate metabolism, polysaccharide degradation, proteolysis, lipid metabolism, and fatty acid breakdown. It is noteworthy that while multiple microorganisms may contribute to the synthesis of the same metabolite, certain key flavor compounds are exclusively generated via specialized metabolic routes.

In the context of targeted flavor modulation, Lu et al. demonstrated that exogenous addition of L-leucine during *Saccharomyces cerevisiae* fermentation of fruit juice enhanced the activity and metabolic flux of key enzymes in the leucine-derived metabolic pathway. This intervention broadly influenced central carbon metabolism, leading to a marked increase in the production of higher alcohols and esters such as isoamyl alcohol and isoamyl acetate [74]. This strategy illustrates a practical means of steering microbial metabolism through targeted nutrient supplementation to intensify desirable flavor traits, offering valuable insights for flavor management in fermented beverages including wine, *huangjiu*, and soy sauce.

In a study on high-salt fermentation of mandarin fish, halotolerant lactic acid bacteria—including *Lactobacillus plantarum*, *Lactobacillus fermentum*, and *Lactobacillus* sakai—were shown to promote the oxidative degradation of phospholipids, particularly phosphatidylcholine, releasing free fatty acids. These polyunsaturated fatty acids subsequently undergo auto-oxidation and enzyme-catalyzed oxidation, yielding a range of volatile aldehydes and alcohols that collectively shape the characteristic fatty aroma and overall flavor profile of the product [75]. These findings offer a systematic strategy for modulating lipid oxidation and optimizing flavor and safety through controlled salinity and microbial community design.

In a novel approach to flavor research in *baijiu*, Xu et al. first screened *Bacillus* strains expressing high levels of *AlsS*/*AlsD* enzymes, then adjusted fermentation conditions and community structure to suppress the activity of functional bacteria (e.g., *Pseudomonas*) carrying *TpdAB* degradation enzymes. This dual strategy effectively reduced the loss of tetramethylpyrazine (TTMP), a key flavor component [76]. By moving beyond a singular focus on biosynthetic pathways, this study established a complete “synthesis–degradation” metabolic network for TTMP within the *baijiu* system, providing a new theoretical basis for understanding the dynamic changes of flavor compounds during fermentation.

### 4.2. Extracellular Polysaccharides Precisely Modulate

Extracellular polysaccharides (EPS) synthesized by LAB are natural macromolecules with significant applications in a wide range of fermented and formulated foods. These biopolymers effectively improve food texture, enhance palatability, and increase water-holding capacity and stability, making them key functional ingredients in the modern food industry. The functional versatility of EPS stems from their diversity in molecular weight, branching degree, and monomer composition, which enables specific interactions with food components such as proteins and starches, thereby modulating the rheological and stability properties of the food matrix [77,78,79,80]. In practical applications, EPS incorporation generally leads to increased viscosity, improved texture, enhanced emulsion stability, and overall optimization of mouthfeel.

The functional role of EPS is particularly evident in fermented dairy products. Neutral EPS produced through microbial metabolism can modulate intermolecular protein interactions, thereby reducing gel hardness. In contrast, negatively charged EPS binds to acidic groups on casein, reinforcing the casein network and significantly increasing the apparent viscosity of the product. Furthermore, high-molecular-weight EPS with rigid backbones and low branching degrees can form entangled polymer networks that enhance product firmness and whey retention capacity. This structure imparts characteristic shear-thinning rheological behavior, thereby comprehensively improving the sensory quality of fermented milk [81].

EPS also show considerable potential for texture modification in non-dairy systems, such as plant-based products including oat milk and soy milk. They enhance system viscosity by increasing steric hindrance and promoting molecular entanglement between proteins. In aqueous environments, the thickening effect of EPS is closely associated with their molecular morphology—such as branching pattern and backbone flexibility—as well as molecular weight and charge distribution. For example, EPS produced by *Lactobacillus plantarum* CSK reduces the shear strain rate in soymilk, improves gel elasticity and recovery, and ultimately contributes to the formation of a more robust gel network [81,82].

Therefore, the functional efficacy of EPS in food texture regulation depends largely on the precise interplay between their molecular properties and specific food matrices. This attribute positions EPS as a valuable tool for targeted texture design, offering broad prospects for future food applications.

### 4.3. Safety Control

The amensalism between different species can effectively inhibit the growth of pathogenic microorganisms, including pathogenic bacteria and pathogenic fungi, thereby enhancing the safety of fermented foods. Bacteriocins are antimicrobial peptides secreted by certain microorganisms to gain ecological competitive advantage. These compounds are harmless to the producer strains but effectively inhibit the growth of competing microorganisms [83]. In an era of increasing concern over the health risks posed by chemical preservatives, bacteriocins have attracted attention as promising natural antimicrobial alternatives. They not only exhibit intrinsic antibacterial activity but can also act synergistically with certain antibiotics [84]. With the escalating challenge of bacterial resistance and growing consumer demand for clean-label foods, the development of novel and safe bio-preservatives has become increasingly urgent.

Studies have shown that LAB isolated from traditional fermented vegetables can produce antimicrobial substances active against a range of common foodborne pathogens, including both Gram-positive and Gram-negative bacteria. This broad-spectrum inhibitory activity highlights the potential of LAB as natural bio-preservatives for controlling pathogens in diverse food matrices. However, the practical application of bacteriocins faces several challenges, such as difficulties in large-scale production and purification, relatively high cost, incomplete cytotoxicity profiles, and a limited antimicrobial spectrum. Moreover, bacteriocins are susceptible to degradation by human proteases, rendering them inactive and thus unsuitable for direct oral administration. Beyond directly inhibiting pathogens, microbial interactions can also enhance the overall safety of fermented foods by degrading or reducing hazardous compounds such as carcinogenic precursors and allergens.

Organic acids represent another crucial class of safety-related metabolites in fermentation systems. They contribute to the characteristic acidity of fermented foods while fulfilling essential preservative functions. The dynamic accumulation of multiple organic acids often results from synergistic metabolism within microbial communities. For example, during kombucha fermentation, yeasts preferentially utilize fructose via glycolysis to produce ethanol, while acetic acid bacteria oxidize glucose at the C-6 position to generate mildly acidic glucuronic acid, simultaneously converting ethanol into acetic acid [85]. In addition to acetic and glucuronic acids, kombucha contains various other organic acids, including citric, malic, tartaric, and succinic acids [67]. The combined presence of these acidic compounds significantly lowers the system pH, effectively suppressing the growth of undesirable microorganisms.

In summary, the current safety assurance framework for fermented foods has begun to incorporate a technical system centered on bacteriocins and organic acids. To further realize their application potential and improve risk management, future research should focus on three key directions: overcoming technical bottlenecks in bacteriocin production and stabilization, elucidating multi-factor regulatory networks in fermentation processes, and enhancing public understanding of fermented food safety. Such efforts will help shift the field from a reactive “post-production preservation” approach toward proactive “end-to-end control,” better aligning with consumers’ enduring demand for safe, natural, and health-promoting foods.

## 5. Utilizing Multi-Omics Research to Explore the Relationship Between Fermented Foods Composed of Complex Microorganisms and Their Functionality, Flavor, and Quality

**Figure 4:**
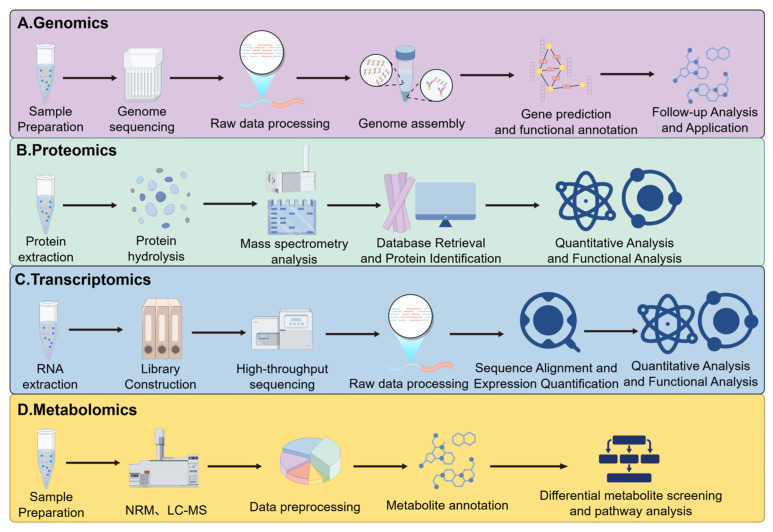
Schematic Diagram of Multi-Omics Integration. (**A**) Genomics: Investigating the structure, function, and mechanisms governing the transmission and regulation of genetic information across all genomes within cells and tissues; (**B**) Proteomics: Study the expression, modification and interactions of all proteins within cells or tissues; (**C**) Transcriptomics: Research into the expression and regulation of all RNA within cells/tissues; (**D**) Metabolomics: Study the composition and dynamic changes of all metabolites in cells/tissues.

In recent years, the application of multi-omics technologies to study microbial interactions in fermented foods has advanced considerably. These approaches deliver comprehensive insights into changes in differentially expressed genes (DEGs), shifts in microbial community structure, protein expression dynamics, and active metabolic pathways throughout fermentation (Table 2), offering valuable perspectives for both scientific and industrial applications. As outlined in Figure 4, integrated multi-omics—a core methodology in systems biology—focuses on consolidating multidimensional data from genomics, transcriptomics, proteomics, and metabolomics. By employing computational tools such as machine learning, this strategy enables deep exploration of the complex linkages between microbial metabolic networks and phenotypic outcomes. It effectively overcomes the constraints inherent in single-omics studies, allowing for a holistic analysis of dynamic fermentation processes. As a result, this approach supports a more systematic understanding of microbial community structure, functional capacity, and the mechanistic basis of product quality, thereby establishing a solid theoretical foundation for optimizing fermentation processes.

### 5.1. Flavor

Fermentation is a biochemical process in which microorganisms—including bacteria, yeasts, and fungi—metabolize carbohydrates, proteins, and lipids to produce a diverse array of metabolites. Among these, flavor compounds play a decisive role in shaping the aroma, taste, and overall sensory characteristics of fermented foods. With the development of systems biology, integrated multi-omics strategies have become powerful tools for deciphering the complexity of this process.

**Figure 5:**
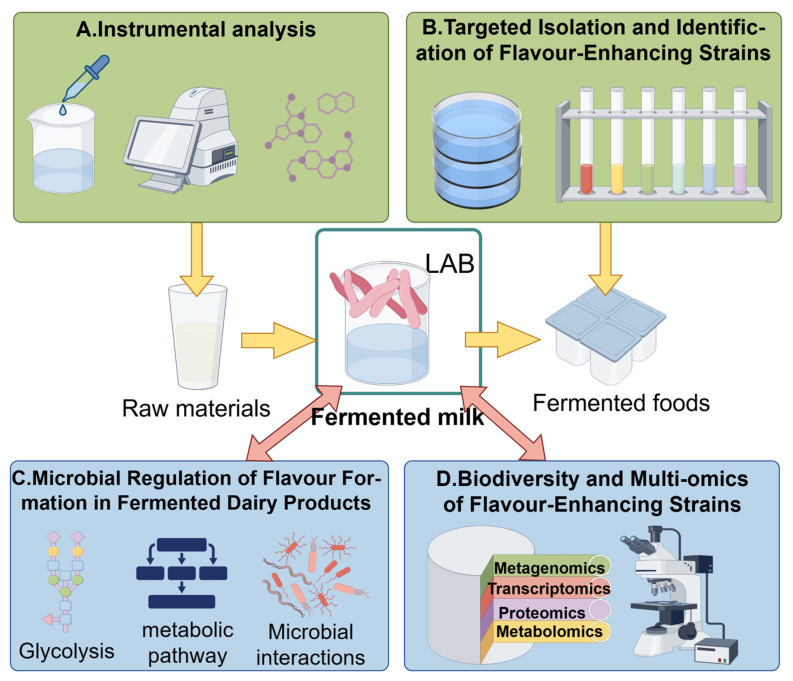
A multi-dimensional technical framework for flavor formation and lactic acid bacteria (LAB) metabolism in fermented dairy products.

In the fermented black bean (*douchi*) system, Wu et al. applied a combined transcriptomic, proteomic, and metabolomic approach to systematically reveal the regulatory relationships between differentially expressed genes and the biosynthesis of secondary metabolites. Their study identified 130 upregulated metabolites and 160 downregulated proteins that collectively contribute to the development of the characteristic flavor profile of *douchi* [89]. Similarly, in *baijiu* fermentation research, the integration of metagenomics and untargeted metabolomics has not only identified core microbial functional units responsible for flavor formation but also delineated the dynamic cooperative networks among diverse microbial communities during fermentation. This provides a systematic framework for achieving precision quality control at the microbial level [86]. In a study on the solid-state batch fermentation of green tea, integrated genomics and metabolomics revealed intrinsic links between dominant fungal communities (primarily *Aspergillus* spp.) and non-volatile flavor compounds. The research confirmed that *Aspergillus* species secrete a wide range of hydrolytic enzymes—such as cellulase, pectinase, and protease—that effectively break down substrates and facilitate the formation of flavor precursors [90]. As illustrated in Figure 5, within dairy fermentation systems, research typically commences with raw materials, systematically tracing the entire process by which lactic acid bacteria drive the synthesis of flavor compounds through metabolic pathways including proteolysis, glycolysis, and lipolysis. This multidimensional research framework encompasses flavor compound analysis, functional strain selection, and the elucidation of microbial regulatory mechanisms. It aligns closely with the intrinsic logic of multi-omics integration, vividly demonstrating the synergistic advantages of this strategy in deciphering flavor formation within fermented foods.

The core value of multi-omics integration lies in its ability to transcend the limitations of single-omics analyses at a systems level. By correlating gene expression, protein function, and metabolite dynamics, it provides a holistic view of the molecular mechanisms underpinning flavor formation [91]. When combined with computational methods such as machine learning and deep learning, researchers can even predict the formation and transformation pathways of key flavor compounds such as esters and aldehydes [92]. Furthermore, cross-validation and complementary data exchange among multiple omics datasets help correct information gaps or biases introduced by individual technologies, thereby enabling more accurate identification of dominant microbial strains, key genes, functional enzyme systems, and their associated characteristic flavor compounds [93].

Predictive models built using these approaches—linking microbial community structure to flavor quality—provide a theoretical basis for the targeted screening of key functional microorganisms and the design of flavor modulation strategies. Further integration of flavoromics with artificial sensory evaluation offers the potential to achieve precise quantification of aroma-active components and scientifically predict flavor profiles [94]. In summary, multi-omics technologies provide a new perspective for systematically understanding the molecular mechanisms of flavor formation in fermented foods. This approach not only holds significant theoretical value but also demonstrates broad application potential in advancing the precision and standardization of the fermentation industry.

### 5.2. Quality

The stability and safety of fermented food quality are often challenged by metabolic variability and complex interactions within microbial communities, making consistent quality assurance difficult to achieve [42]. Multi-omics technologies offer powerful means to systematically analyze the roles of beneficial, pathogenic, and spoilage microorganisms throughout fermentation. The relative stability of fermented products generally depends on effective suppression of pathogens through factors such as raw material selection, fermentation duration, and pH control.

The use of starter cultures from different sources—such as the successful fermentation observed with kimchi or garlic, compared to unsuccessful outcomes with ginger or red chili—leads to significant differences in dominant microorganisms and their metabolite profiles. This underscores the critical influence of microbial community composition on final product quality [95]. Similarly, Medina et al. reported that although green olives maintained good quality in early to mid-fermentation stages, the later emergence of multiple spoilage microorganisms negatively affected product integrity [96].

Integrated proteomic and transcriptomic analyses further revealed that when the pH of fermented milk dropped to 5.5, expression of glutamate-related proteins was suppressed, whereas key enzymes involved in cystine catabolism were significantly upregulated, directly influencing dairy quality attributes [97]. These cases collectively demonstrate that the dynamic succession of microbial communities is a decisive factor in determining the quality of fermented products.

Thus, multi-omics technologies have become essential tools in modern food biotechnology, providing a robust scientific basis for improving fermentation quality, enhancing safety, and optimizing microbial interactions.

### 5.3. Functionality

Multi-omics integration strategies are increasingly recognized as powerful methodologies for systematically deciphering the functional components of fermented foods. By synthesizing biological information across multiple dimensions, these approaches significantly improve the accuracy of bioactive compound identification while providing systematic insights into their formation mechanisms and safety attributes.

In the field of bioactive peptide research, scientists have integrated peptidomics with metagenomics to conduct in-depth analysis of fermented soybean systems. These investigations led to the identification of 714 natural peptides—561 of which were novel—and specifically revealed that certain peptide fragments derived from the degradation of soybean allergenic proteins (e.g., IPPGVPY and PLDLTSFVLHEAI) exhibit significant monoamine oxidase inhibitory activity. This finding provides molecular-level evidence of how microbial fermentation converts allergenic proteins into neuroprotective bioactive peptides [98].

Regarding antioxidant metabolites, the combined application of transcriptomics and metabolomics has clarified the antioxidant mechanisms of lactic acid bacteria. Studies show that under aerobic conditions, lactic acid bacteria upregulate the expression of genes such as superoxide dismutase while simultaneously synthesizing metabolites including extracellular polysaccharides and short-chain fatty acids. These components act synergistically to enhance the system’s free radical scavenging capacity, an antioxidant pathway validated in both fermented dairy products and traditional foods such as fermented eggs [99].

Compared to the systems-level perspective provided by multi-omics, traditional single-omics analyses (e.g., genomics alone) exhibit notable limitations. Although genomics can reveal changes in gene abundance and suggest potential microbial interactions, it struggles to establish direct links between genetic potential and functional phenotypes [100]. The strength of multi-omics integration lies in its ability to trace the genetic origins of specific bioactive compounds [101] and dynamically map phenotypic outcomes through integrated transcriptomic and proteomic data, thereby advancing our holistic understanding of complex fermented food systems.

By systematically integrating metagenomic, transcriptomic, proteomic, and metabolomic data, researchers can reconstruct comprehensive microbial metabolic networks. This enables precise identification of key functional genes and their regulatory patterns, ultimately supporting targeted control of fermentation processes. This strategy not only deepens scientific understanding of fermented food functionality but also establishes a robust foundation for the industrial production of high-quality functional fermented foods.

In summary, multi-omics technologies have become indispensable for deciphering bioactive components in fermented foods. Through integration of biological information across multiple levels, they comprehensively reveal the formation mechanisms of functional components during microbial fermentation, providing a solid theoretical and technical basis for the targeted design and quality improvement of functional fermented products.

### 5.4. Industrial Applications

In recent years, the understanding of microbial succession mechanisms in food fermentation has significantly advanced, with related research outcomes increasingly being implemented in industrial settings. This progress is reflected in several key areas, including the establishment of dedicated microbial resource banks for fermented foods, the development of functional prediction models, and improved optimization and control of fermentation processes. Together, these developments provide strong support for the standardization and quality enhancement of traditional fermented products. In particular, omics technologies are demonstrating unique value in industrial applications for process prediction and optimization.

In Korean kimchi production, for instance, the Fermentation Microbiome Database (ODFM) integrates multi-omics data from the World Kimchi Institute, establishing a foundational resource for screening starter cultures with superior fermentation performance and safety attributes. This database is expected to enable predictive modeling and precise regulation of fermentation processes in the future, thereby advancing the standardization of kimchi production [102]. In the context of *baijiu* manufacturing, multi-omics approaches have systematically clarified the role of microbial communities in cellar mud in shaping key flavor compounds. Researchers successfully established a correlation model between microbial succession in artificial cellar mud and flavor metabolism. Functional predictions indicated that prokaryotic microorganisms in specific batches of cellar mud were significantly involved in organic acid metabolic pathways—a finding later corroborated by proteomic and metabolomic data. These key microbes, proteins, and metabolites now serve as effective biomarkers for evaluating cellar mud quality and monitoring fermentation status, offering clear targets for optimizing mud cultivation and improving *baijiu* quality [103].

Notably, emerging gene editing technologies are creating new opportunities in fermented food science. As highlighted by Pan and Barrangou, CRISPR-based genome editing enables precise genetic modification of food-associated microorganisms. This technology not only facilitates the development of enhanced probiotics and novel biotherapeutics but also allows targeted modulation of microbial community structures within food matrices, demonstrating considerable application potential [104,105].

With the deepening integration of multi-omics technologies and gene-editing tools, research in food fermentation is shifting from descriptive analysis toward mechanistic insight and precision control. These advances not only enhance the understanding of microbial functions in traditional fermentation but also establish a solid foundation for standardized production, quality improvement, and innovative development of fermented foods.

## 6. Conclusions and Future Perspectives

This review systematically examines the composition, dynamic succession patterns, and regulatory mechanisms of microbial communities in fermented foods, with a focus on how microbial interactions govern flavor, texture, and safety. Studies indicate that core functional microorganisms—primarily lactic acid bacteria, yeasts, and molds—collectively shape the distinctive qualities of fermented foods through synergistic metabolic activities, including carbohydrate metabolism, proteolysis, and lipid degradation. Within these processes, mutualistic and competitive relationships critically influence the direction and intensity of key metabolic pathways.

In recent years, the widespread adoption of integrated multi-omics approaches—such as metagenomics and metabolomics—has significantly advanced our understanding of the relationships between microbial communities and volatile flavor compounds, nutritional constituents, and bioactive metabolites. These techniques provide novel perspectives and methodological support for the targeted regulation of fermented food quality. Nevertheless, several critical challenges remain. First, functional annotation systems for microbial genes are still incomplete, with considerable gaps in knowledge regarding non-model species and complex secondary metabolites. Second, the high cost of multi-omics technologies and the complexity of data analysis limit their large-scale industrial application. More importantly, the lack of unified data standards across research platforms impedes cross-study comparisons and meta-analyses, hindering the broader adoption and development of these methodologies in the fermented food sector.

Future efforts should prioritize the following directions: developing specialized databases for fermentative microorganisms to improve gene functional annotation, with an emphasis on characterizing genes of unknown function; establishing cost-effective, small-sample multi-omics workflows to facilitate routine quality monitoring; implementing cross-platform data standardization protocols to enhance reusability and integration; and strengthening causal validation between microbial interactions and metabolic outcomes to advance from correlation to mechanism.

By systematically addressing these challenges, multi-omics technologies can help transition fermented food production from an experience-based practice to a mechanism-driven industry, supporting standardization, functionalization, and sustainable development. The theoretical framework and technical pathways outlined in this review are intended to provide systematic guidance for understanding quality formation mechanisms and developing enhancement strategies, offering both scientific value and practical relevance.

## Figures and Tables

**Table 1:** Types of Microbial Metabolism.

Metabolic Type	Specific Metabolic Pathways	Metabolic Microorganism
Glycometabolic pathway	Glycolysis (EMP pathway)	*Escherichia coli*, *Saccharomyces cerevisiae*, *Bacillus subtilis*, *Lactobacillus*
	Tricarboxylic acid cycle (TCA cycle)	Aerobic/Facultative aerobic microorganisms (*Escherichia coli*, yeast, *Acetobacter*)
	Pentose phosphate pathway	Yeast, *Escherichia coli*, cyanobacteria, actinomycetes
	Acetaldehyde cycle	*Escherichia coli*, rhizobia, certain fungi (such as *Aspergillus niger*)
Lipid and Fatty Acid Metabolism	Fatty acid synthesis	Yeast, *Escherichia coli*, Actinomycetes, plant pathogenic fungi (such as *Fusarium*)
	Fatty acid β-oxidation	Aerobic microorganisms (*Escherichia coli, Pseudomonas*) Facultative anaerobic microorganisms (*Saccharomyces cerevisiae*)
	Glycerol metabolism	*Escherichia coli*, yeast, lactic acid bacteria
Polysaccharide degradation	Starch hydrolysis	*Bacillus subtilis*, *Aspergillus niger, Aspergillus oryzae*, *Rhizopus*
	Cellulose decomposition	*Mucor*, cellulose-degrading bacteria (such as *Clostridium thermofibrinolyticum*), certain actinomycetes (such as *Streptomyces*), and the gut microbiota of termites
	Pectinase	*Aspergillus niger*, *Bacillus subtilis*, *Bacillus pectoralis*
	glycogenolysis	*Escherichia coli,* yeast, certain bacteria (such as streptococci)

**Table 2:** Research Findings on Fermented Foods via Multi-Omics Approaches.

Fermented Foods	Multi-Omics Approaches	Significant Findings	References
Fermented soybeans	Transcriptome analysis and proteome analysis	Main metabolic pathways involved carbohydrates, proteins, and amino acids.	[64]
Fermented fish	Transcriptomics and Metabolomics analyses	During microbial interactions, *Saccharomyces cerevisiae* and *Lactobacillus plantarum* produce antibiotics that influence carbohydrate and energy metabolism.	[86]
Light-flavor Baijiu	Metagenomics and Metabolomics	alcohols and esters were the most abundant metabolites.	[65]
Blood orange wine	volatilomics, genomics, and transcriptomics	Enhanced the complexity and appeal of the aroma; a substantial portion of the *P. kudriavzevii* BP15 genome is dedicated to carbohydrate, amino acid, and energy metabolism.	[87]
Fermented Milk	Metagenomics and Metabolomics	Potential Mechanism Underlying the Hypotensive Effect of *Lactobacillus plantarum* SR37-3 (PFM-SR37-3) Fermented Milk in Spontaneously Hypertensive Rats (SHR)	[88]

## Data Availability

No new data were created or analyzed in this study. Data sharing is not applicable to this article.
